# Preparation and Characterization of Eugenol Incorporated Pullulan-Gelatin Based Edible Film of Pickering Emulsion and Its Application in Chilled Beef Preservation

**DOI:** 10.3390/molecules28196833

**Published:** 2023-09-27

**Authors:** Zhi-Gang Ding, Yi Shen, Fei Hu, Xiu-Xiu Zhang, Kiran Thakur, Mohammad Rizwan Khan, Zhao-Jun Wei

**Affiliations:** 1 School of Food Engineering, Anhui Science and Technology University, Fengyang 233100, China; 2 School of Biological Science and Engineering, Ningxia Key Laboratory for the Development and Application of Microbial Resources in Extreme Environments, North Minzu University, Yinchuan 750021, Chinahufei@hfut.edu.cn (F.H.); kumarikiran@hfut.edu.cn (K.T.); 3 School of Food and Biological Engineering, Hefei University of Technology, Hefei 230009, China; 4 Department of Chemistry, College of Science, King Saud University, Riyadh 11451, Saudi Arabia; mrkhan@ksu.edu.sa

**Keywords:** eugenol, pullulan–gelatin, Pickering emulsions, chilled beef

## Abstract

The purpose of this study was to develop a composite film composed of eugenol Pickering emulsion and pullulan–gelatin, and to evaluate its preservation effect on chilled beef. The prepared composite film was comprehensively evaluated in terms of the stability of emulsion, the physical properties of the film, and an analysis of freshness preservation for chilled beef. The emulsion size (296.0 ± 10.2 nm), polydispersity index (0.457 ± 0.039), and potential (20.1 ± 0.9 mV) proved the success of emulsion. At the same time, the films displayed good mechanical and barrier properties. The index of beef preservation also indicated that eugenol was a better active ingredient than clove essence oil, which led to the rise of potential of hydrogen, chroma and water content, and effectively inhibited microbial propagation, protein degradation and lipid oxidation. These results suggest that the prepared composites can be used as promising materials for chilled beef preservation.

## 1. Introduction

The consumption of chilled beef has increased significantly due to its superior flavor and better meat quality, water-holding capacity and nutritional value [1]. Due to its highly nutritious nature [2], microbial pollution, oxidative deterioration, and even peculiar smells and discoloration can occur during processing and storage [3]. Although frozen storage techniques can improve the shelf life of beef, it is clear that the decline in quality during the freezing process always affects the taste and nutrition of the products [4]. In addition, traditional cold fresh technology can only ensure the freshness of beef in a short time. There is a growing concern about the health and hygiene of chilled beef products. Therefore, the methods used to extend the shelf life of chilled or fresh beef have been key concerns in recent years [5,6]. Low-temperature preservation technology is effective in keeping the meat fresh, but it is not enough to maintain a relatively long shelf-life. Therefore, other methods are required to extend the shelf life.

Recently, some methods related to guaranteeing the quality and prolonging the consumption life of meat products were reported, among which packaging is the most effective. The combination of active substances and packaging materials provides several functional characteristics that do not exist in the traditional packaging system, and can overcome the problems of traditional packaging [7]. Natural compounds such as plant essential oils are considered to be safe and environmentally friendly functional preparations [8,9], which has confirmed their potential use in food packaging [10,11]. More excitingly, research has demonstrated that adding essential oils to edible films used in meat packaging is more effective than addition them directly to the meat product. Essential oils added into edible films can selectively and sustainably migrate from the packaging to the surface of the meat product, thus maintaining high concentrations in the parts where they are most needed [12].

Eugenol (Eu), a natural phenolic compound, is the main component of clove essential oil [13], which displays strong insecticidal, bacteriostatic, and antiseptic effects [14,15]. It is also a food additive allowed by the European Union, the United States, and China [16]. More recently, it was reported that Eu could be combined with polymers for further innovations in active packaging to effectively improve the quality and shelf life of meat [17,18], and be added to the formula of the film for food preservation by preventing oxidation reactions [19]. Previous studies also showed that Eu exerted its antioxidant effect on the surfaces of products by increasing the non-specific permeability of the microbial cytoplasmic membrane, thereby disrupting the cell membrane and causing the leakage and death of cell contents [20]. However, the poor water solubility of Eu prevents its application in the field of the preservation of meats [18,21]. In this context, Pickering emulsion (PE), prepared by solid particle stabilization, offers new opportunities for the application of Eu in the preservation of meats. The Eu-incorporated edible film with a Pickering emulsion exhibited the mechanical properties of high density, low water content and low permeability, as well as good water barrier properties and significant antioxidant activities [22]. PE encapsulation can cause Eu to be more evenly dispersed in the membrane solution, which in turn improves the low water solubility, high volatility and weak stability, and enables greater bioactivity [23]. Therefore, Eu loading in Pickering emulsion has become a promising method to prevent the evaporation loss of Eu, minimize the unfavorable effects of on food flavor, and enhancing the antibacterial and antioxidant properties of Eu during food preservation.

In the present study, a polymer matrix based on pullulan–gelatin was chosen to be combined with a picolinic emulsion containing Eu to prepare novel composite films. To our knowledge, the application of Eu as a preservative in meat products is a relatively new research area that is worth exploring. Therefore, this study intends to study the applicability of Eu as an active ingredient in the fresh-preserving films of beef. Firstly, a series of characterizations of emulsions and films was prepared. Then, the potential values of hydrogen (pH), color, moisture content, thiobarbituric acid reactive substances (TBARS), total volatile base nitrogen (TVB-N) and total bacterial colony of the chilled beef were determined. The results of the present research provide a basis for applying the Eu-incorporated pullulan–gelatin-based edible film of Pickering emulsion in the preservation of meats.

## 2. Results and Discussion

### 2.1. Analysis of Emulsion

**Figure 1:**
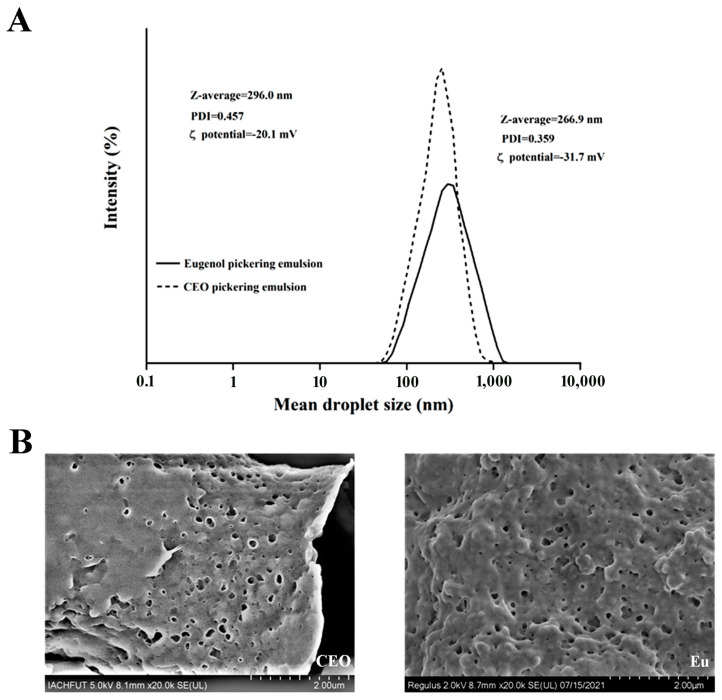
The characterization of the PEs containing Eu and CEO. (**A**) The particle size distribution, the polydispersity index, and the ζ-potential distribution of the PEs containing Eu and CEO. (**B**) The microstructure of PEs powder containing Eu and CEO. As shown in the graph, the bar is equal to 2.00 μm.

The particle size, PDI and zeta potential data of Pickering emulsions containing clove essential oil (CEO) [24] and Eu are displayed in Figure 1A. The mean particle size of the Pickering emulsion added to Eu was 296.0 ± 11.2 nm, the PDI value was 0.457 ± 0.039, and the absolute value of zeta potential was 20.1 ± 0.9 mV. In a study, Li et al. prepared an Eu nanoemulsion containing limonin (an antimicrobial component from citrus seeds), which showed that the mean particle size of Eu nanoemulsions was 213.7 nm, and presented a larger particle size of 245.7 nm with the addition of limonin [25]. In another study, a sub-micron injectable emulsion of Eu was developed with an average particle size of 176.1 ± 10.3 nm and a potential of −40.2 ± 1.8 mV, which showed excellent stability and safety compared to Eu solutions [26]. Compared with a Pickering emulsion containing CEO, the emulsion loaded with Eu showed larger average particle sizes and distributions, and a smaller potential absolute value. The enlargement in the size of the PE loaded with Eu may be due to the aggregation of macromolecular WPI and inulin in the surface layer of the emulsion. Overall, the size of the Eu-loaded PE was similar to that reported for other film formulations using Pickering emulsions [7]. A study showed that the droplet size of orange peel essential oil nanoemulsions became smaller with increasing sonication time and intensity. It is worth noting that high intensities can also lead to agglomeration, which in turn leads to larger sizes. Therefore, nanoemulsions with physical stabilization have wider practical applications [27]. Besides this, the microstructure of the PE containing Eu was consistent with that of the PE containing CEO after freeze-drying (Figure 1B).

### 2.2. Analysis of FT-IR

**Figure 2:**
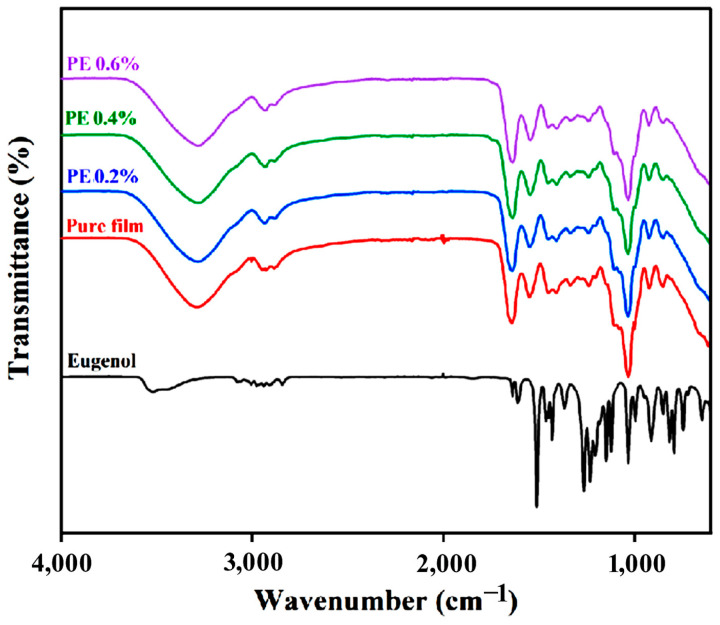
FTIR spectra of different samples. The samples include eugenol, pure film, 0.2% PE, 0.4% PE, and 0.6% PE, respectively. (Pure film, 0.2% PE, 0.4% PE, and 0.6% PE).

The addition of PE loaded with Eu did not significantly change the chemical structure of the pullulan–gelatin film, but weakened some hydrogen bonds in the matrix (Figure 2) [28]. Prulan–gelatin films showed O-H, C-H, -OH and C-O-C stretching vibrations at the absorption peaks of 3327 cm^−1^, 2936 cm^−1^, 1637 cm^−1^ and 1036 cm^−1^, respectively [24]. The peaks at 2924 cm^−1^ and 2856 cm^−1^, corresponding to the methylene symmetric and asymmetric stretching vibration of C/H of CH_3_ and CH_2_, increased in intensity due to a sharp increase in C-H content after loading with Eu, as reported by Zheng et al. [29]. Liao et al. prepared interfacial crystalline oil gel emulsions, and the absorption peaks of the samples at 2920 cm^−1^, 2851 cm^−1^ and 721 cm^−1^ were from the asymmetric, symmetric telescopic vibration and wobble vibration peaks of -CH_2_, respectively, which indicate the presence of van der Waals forces in all the samples [30]. Samples containing emulsions and nanoemulsions showed looser bands for the peaks at 3000–2800 cm^−1^, indicating the better stability of the components added into the edible membrane matrix [31]. At the same time, the C-C vibration peak at 1634 cm^−1^ originated from the C-C stretching vibration of the aromatic ring, which further verifies the successful embedding of Eu. In addition, small peaks were observed to become flatter in the spectra. These changes suggest that the addition of Eu to the films caused some interactions between the functional groups, possibly giving rise to hydrogen or other covalent bonding.

### 2.3. Analysis of Scanning Electron Microscope in Cold Field

**Figure 3:**
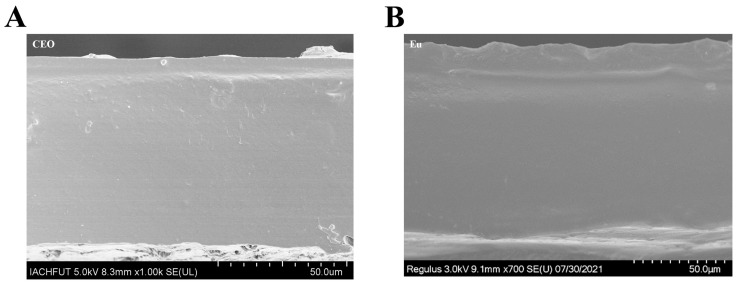
Cold field emission scanning electron microscope images of cross sections of different composite films. (**A**) CEO, (**B**) Eu. As shown in the graph, the bar is equal to 50.0 μm.

The cross section of the film containing PE was dense and the surface showed large protrusions (Figure 3). The addition of Eu to PE led to a coarser surface, which was associated with aggregates or droplets migrating to the top of the film during film drying, resulting in an irregular surface. In this regard, there are many studies wherein the surface roughness of films containing Pickering emulsions has shown varying degrees of increase with the addition of clove [24], marjoram [7], cinnamon [32] and oregano [33] essential oils, as well as chitosan [34] and polylactic acid [35].

### 2.4. Thermogravimetric Analysis

**Figure 4:**
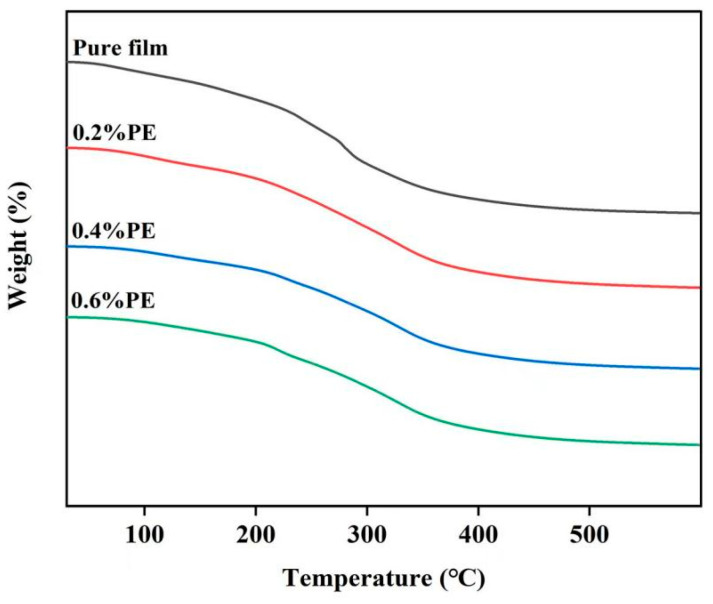
Thermogravimetric curves of different film samples. The samples include pure film, 0.2% PE, 0.4% PE, and 0.6% PE, respectively.

The results of the thermogravimetric analysis are presented in Figure 4. The pure film showed two main stages of weight loss, the first stage between 40 °C and 180 °C, as a result of the evaporation of water from within the film, and the next at 180–500 °C, which was related to the thermal decomposition of the polymer. As it can be seen, three phases were observed in the films containing PE-loaded Eu. The weight loss between 40 and 210 °C was mainly due to residual water and a small proportion of the components in the volatile Eu. There was a complex process at around 210–350 °C involving chain depolymerization and the decomposition of non-volatile components [29]. The last phase of weight loss occurred after 350 °C and was related to the decomposition of Eu, which was due to the successful loading of Eu into the film matrix. This result maintained a similar trend to data obtained in previous studies [24,36]. In addition, there was no obvious difference in mass retention between films with different concentrations of PE, which suggests that the retardation was independent of the amount of Eu and was mainly influenced by temperature. Furthermore, the mass retention was stabilized with the increasing temperature. After reaching 460 °C, no significant difference was seen in the mass retention among all the films [37]. It is worth noting that pullulan–gelatin films have slightly higher mass retention than other films containing PE-loaded Eu. Therefore, PE had a small effect on the thermal stability of the pullulan–gelatin film matrix.

### 2.5. Analysis of Physical Properties of Thin Films

#### 2.5.1. Analysis of Thickness

Thickness was a useful parameter for studying the mechanical properties and water resistance of films. As exhibited in Table 1, the thickness of pure pullulan–gelatin film was 168.0 ± 3.29 μm, while the addition of PE loaded with Eu significantly decreased the thickness index of the films (0.2% PE: 115.7 ± 5.16 µm; 0.4% PE: 101.3 ± 6.52 µm; 0.6% PE: 88.5 ± 2.33 µm). The effect of PE on film thickness significantly increased (*p* < 0.05) as the Eu concentration increased, with the lowest thickness of 88.5 ± 2.33 µm for films loaded with 0.6% PE emulsion. The enhancement in emulsion composition disturbed the ordered structure of the films, leading to a reduction in the solids content of the film matrix, which in turn affected the film thickness. Similar results were reported in previous studies [24,32,38]. On the other hand, a number of studies also reported different results. By adding marjoram essential oil-loaded PE, the thickness values of pectin film samples showed a tendency towards significant increases [7], as the addition of PE could increase the dry matter content and alter the structures of pectin-based films. The thickness of the cellulose films also increased with the addition of oregano essential oil-loaded PE, which was explained by the overlapping structure of the original fibers leading to a discontinuity in the arrangement of the cellulose films [33]. Thus, the trend and the degree of changes in film thickness were also influenced by the different pristine film matrices.

#### 2.5.2. Analysis of Mechanical Properties

The mechanical parameters can effectively describe the mechanical strength of the film and are directly related to its structure. As can be seen in Table 1, TS (pure film: 2.47 ± 0.21 MPa; 0.2% PE: 2.33 ± 0.15 MPa; 0.4% PE: 2.54 ± 0.17 MPa; 0.6% PE: 2.82 ± 0.25 MPa) did not change significantly after adding low concentration emulsion, and elongation at break (0.4% PE: 12.46 ± 1.29%; 0.6% PE: 10.77 ± 0.81%) decreased with increasing TS for the 0.4% and 0.6% PE films. The interplay between polymer matrix and PE interface increased the cohesion of the polymer matrix, caused the formation of high mechanical resistance, and finally reduced the chain sliding during film stretching [24]. However, there are also studies showing the effect of the presence of emulsified oils in the membrane matrix on decreasing TS and enhancing EB. The increase in EB indicated that the addition of emulsified oils resulted in a more tensile and elastic film. This may be due to the penetration of fatty acids through the chains, which reduces the intramolecular interactions between the chains, thus making the chains easier to move [31,39,40]. The addition of a high concentration of PE had a significant effect on elongation at break and tensile strength (*p* < 0.05). However, the developed composite films still showed good mechanical strength and flexibility.

#### 2.5.3. Analysis of Water Vapor Permeability

The water vapor transmittance of the composite membrane is shown in Table 1. The results indicate that the water vapor permeability (×10^−7^ g·m/m^2^·Pa·s) gradually decreased with the increase in Eu content (0.2% PE: 7.17 ± 0.46; 0.4% PE: 6.52 ± 0.33; 0.6% PE: 6.04 ± 0.18) compared with that of the pure film (9.43 ± 0.32). The water vapor transfer process in the film species was influenced by the hydrophilic/hydrophobic ratio of the film, which was related to the solubility of the water molecules, in addition to the heterogeneity of the dispersed phase. The increase in water vapor transmittance was attributed to the interaction between hydrophobic substances and membrane components, which makes the polymer matrix more open to the transportation of water molecules [7]. The existence of Eu reduced the water vapor transmittance of the film because it increased the hydrophobic fragment and water molecular resistance of the film, and it promoted the increase in matrix hydrophobicity and the distortion factor of mass transfer [5].

#### 2.5.4. Analysis of Chromaticity

**Figure 5:**
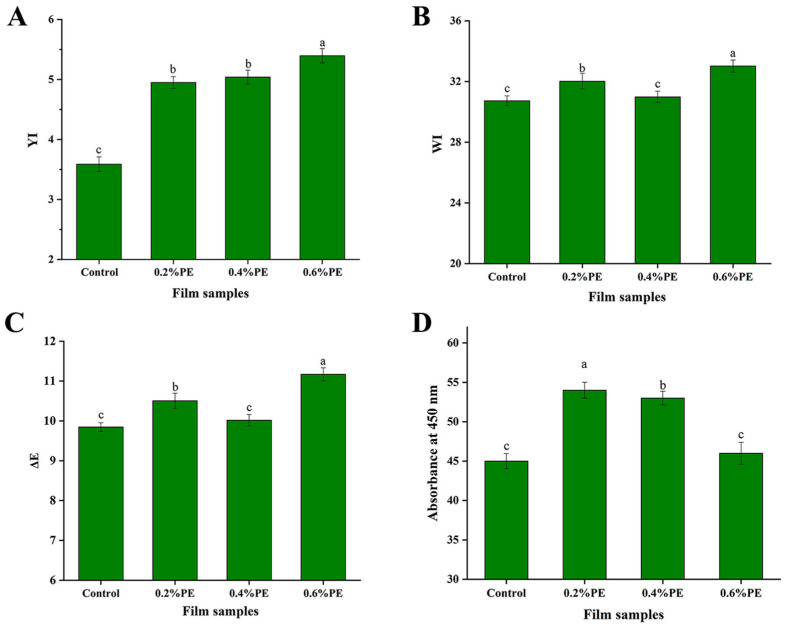
Chromaticity diagram of different film samples. (**A**) YI, (**B**) WI, (**C**) ΔE, (**D**) transmittance. The samples include pure film, 0.2% PE, 0.4% PE, and 0.6% PE, respectively. Data are shown as mean ± SD from three replications and different letters represent a significant difference (*p* < 0.05).

The chromaticity results of the composite film are shown in Figure 5. The chromaticity of the composite film was influenced by PE with higher particle sizes, natural white inulin and bright yellow WPI. The color of Eu itself also changed the color of the film and increased the total color difference. The light scattering effect of Eu dispersed in the film made it transparent and increased the barrier performance against light.

### 2.6. Analysis of pH Value of Chilled Beef

**Figure 6:**
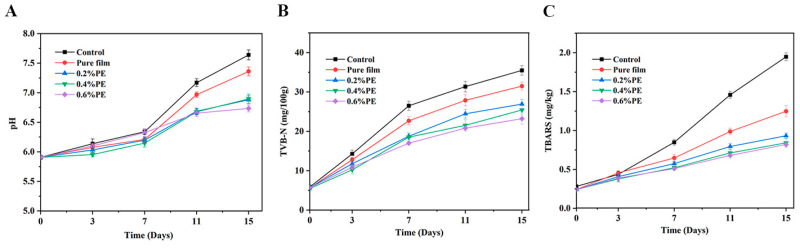
Changes in the pH (**A**), TVB-N (**B**), and TBARS (**C**) values of different film-preserved beef samples at different times. The films include pure film, 0.2% PE, 0.4% PE, and 0.6% PE, respectively. The chilled beef was used as a control.

The initial pH value of meat samples was 5.81, and the pH of all samples increased in varying degrees during storage (control: 7.64; pure film: 7.36; 0.2% PE: 6.88; 0.4% PE: 6.90; 0.6% PE: 6.73) (Figure 6A). The pH of meat samples from the composite film treatment group was slightly lower than that in the control group, which was not obvious (*p* > 0.05). The change trend was consistent with that seen in the pullulan–gelatin film treatment group of PE containing CEO. Previous reports indicate that lactic acid and other acidic substances produced by glycolysis in the initial stage slow down the increase in pH, and the decomposition of protein in the later stage leads to a large accumulation of alkaline components and an increase in pH [5].

### 2.7. Analysis of Chromaticity of Chilled Beef

In the process of fat oxidation, hydroperoxide was destroyed, which promoted the formation of low-molecular weight carbonyl compounds and alcohol compounds, which caused changes in the beef color. Table 2 summarizes the L* value, b* value and a* value of chilled beef in different treatments. The improved meat brightness can be related to high myofibril degeneration [41]. The results show that the L* value was directly proportional to the extension of storage time. In the early stage of storage, the film treatment group containing Eu showed a lower L* value than the control group. The use of Eu film could reduce the denaturation of protein in meat, reduce the exposure of hydrophobic groups and water loss, change the refractive index of the beef surface and effectively prevent beef from turning white [42]. However, with time, the water was evaporated and the brightness was decreased. The yellow substance produced by protein denaturation and fat oxidation would increase the B* value [43]. The addition of Eu inhibited bacterial proliferation and reduced the oxidation degree of protein and fat. During the whole storage period, the reddish brown ferri-myoglobin increased and the a* value decreased with storage time. After storage for the 10th day, the a* value of the control group revealed a downward trend compared to the three essential oil treatment groups (*p* < 0.05). This was because the antioxidant activity of Eu hindered the oxidation of myoglobin and delayed the color change of meat. The fresh-keeping ability of composite films and the slow release of Eu may be enhanced by the protective effect of PE.

### 2.8. Analysis of Moisture Content of Chilled Beef

The moisture content in beef is one of the most important indicators when consumers buy, so it was necessary to measure its change. The water contents of beef after different composite film treatments are displayed in Table 3. All the samples showed a downward trend in moisture content during storage, which was due to the oxidation of protein in myosin, and the cross linking of myosin to produce stable aggregates resulting in the contraction of muscle fibers and the decrease in moisture content. Compared with the control group (75.3 ± 1.2% to 76.3 ± 1.1%), the pure film and the film containing Eu treatment group showed a decreasing trend over time, especially the film containing Eu. The reason may be that gelatin and pullulan polysaccharide have certain water absorption properties, and can absorb water in food. In addition, the composite film combined with Eu could reduce the water vapor transmission coefficient and alleviate the water loss in beef [5]. At the same time, it could reduce microbial activity, prevent myosin degeneration, and effectively reduce water loss due to the bacteriostatic and antioxidant effects of Eu.

### 2.9. Analysis of Total Volatile Base Nitrogen (TVB-N) Value of Chilled Beef

TVB-N was widely used to evaluate the freshness of meat products. A high TVB-N value means that the meat is seriously deteriorated. The increase in TVB-N is due to the decomposition of the protein by spoilage bacteria and enzymes on the surface of chilled beef, resulting in ammonia, dimethylamine and trimethylamine. The TVB-N value showed an increasing trend, which was consistent with the performance of composite films containing CEO with increasing storage times (Figure 6B). In the first 7 days, the TVB-N values of all the samples significantly increased (*p* < 0.05), and then the trend slowed down. The samples coated with composite film containing Eu showed significantly lower values than those from the control and pure membrane treatment groups. In the previous study, this was due to the excellent bacteriostasis of phenols, which could combine with proteins at multiple points so as to destroy microbial cell films and effectively inhibit the growth of bacteria [21]. At the same time, it had a strong enzyme inhibition effect, which could have limited the decomposition of proteins and fat by bacteria and slowed down the increase in TVB-N. Moreover, Eu and pullulan–gelatin film had synergistic effects, and showed excellent inhibitory effects [44].

### 2.10. Analysis of Thiobarbituric Acid (TBARS) Value of Chilled Beef

The TBARS value can be used to assess the degree of lipid oxidation of meat. It is the measurement standard of the malondialdehyde content of fatty acid oxidation degradation products in meat products. It causes lipid peroxidation when the fat in beef encounters oxygen. Food rancidity, taste changes, peculiar smells, and even food poisoning can lead to lipid peroxidation. The change in TBARS value during the storage of chilled beef is shown in Figure 6C. It can be seen that the TBARS value of the initial beef was 0.26 mg/kg. The TBARS values of the composite film treatment group and the control group improved considerably with the storage time. The increasing trend exhibited by the Eu composite film treatment group decreased significantly (*p* < 0.05). On the one hand, gelatin and pullulan polysaccharide could form a dense protective film layer on the surface of beef, and the low oxygen permeability could effectively block the external air, especially controlling the entry of oxygen [45]. On the other hand, this was because Eu, which was the main component of CEO, had excellent antioxidant properties that could chelate with metal ions to inhibit the free radical chain reaction. This could effectively delay the lipid oxidation of beef, thereby slowing the rate of beef fat oxidation [46]. Meanwhile, PE protected the active substances of Eu and perpetuated its antioxidant effect [18].

### 2.11. Analysis of Total Bacterial Count of Chilled Beef

The total number of colonies was used to evaluate the quality of meat products. The growth trend of microorganisms in the initial stage of storage was relatively slow, as shown in Table 4. The total number of colonies in chilled beef increased significantly with the extension of storage time (*p* < 0.05). The initial bacterial count of chilled beef was 4.25 log CFU/g. The colony number of meat samples in each treatment group showed an upward trend with time. The control group increased the fastest, exceeding the microbial limit on the 7th day and reaching 9.43 log CFU/g on the 11th day. Pure pullulan–gelatin may have certain bacteriostatic effects in the early stage because of its ability to control oxygen content, and there was no significant difference between the later stage of storage and the control group (*p* < 0.05). The total number of colonies in the treatment group containing Eu showed no significant difference (*p* > 0.05) from the concentration of Eu during storage. These results show that composite films containing Eu could inhibit microbial growth during beef preservation to a certain extent, delay the activity time of Eu, achieve the effect of bacteriostasis and preservation, and prolong the shelf life of beef [8]. Eu, as the main active ingredient in clove essential oil, contains active phenolic hydroxyl groups that could be combined with amino or carboxyl groups in protein molecules. A previous study claimed that the hydrophobic benzene ring structure could also hydrophobically bind to protein molecules, and this multipoint binding could efficiently prevent bacterial infection [13,14].

## 3. Materials and Methods

### 3.1. Materials

Fresh beef was sourced from a local market in Hefei, Anhui province. The gelatin, pullulan polysaccharide, inulin, and whey protein isolate were of food grade quality (Source Leaf Biology (Shanghai, China)). Eu, sodium chloride, sodium hydroxide, hydrochloric acid, boric acid, trichloroacetic acid, potassium bromide, absolute ethanol and PCA medium were of analytical grade (Sinopharm Chemical Reagent Co., Ltd. (Shanghai, China)).

### 3.2. Preparation of Pickering Emulsion

The Pickering emulsion (PE) was carried out as described by Shen et al. [24]. For the biopolymer suspension, the whey protein isolate (WPI) and inulin were dissolved in distilled water, and then magnetically stirred at 25 °C for 24 h. Then, Eu (0.5 wt. %) was slowly transferred into the suspension, and the mixed solution was cut (7500 g) to ensure that Eu was encapsulated in the WPI/inulin mixture.

### 3.3. Characterization of Emulsion

The particle size of the PE-loaded Eu was obtained at 25 °C by dynamic light scattering using a particle size analyzer (Zetasizer Nano-ZS, Malvern, UK), including zeta potential and polydispersity index (PDI). The emulsion samples were diluted with ultrapure water in the appropriate multiplicity before the test was performed to avoid multiple scattering effects. The PE structure loaded with Eu was observed using a cold field emission-scanning electron microscope (FE-SEM) (SU8020, Hitachi, Japan).

### 3.4. Preparation of Films

Pullulan–gelatin films were prepared following the description of Shen et al. [24]. Firstly, gelatin was dissolved in the water preheated at 90 °C, followed by the addition of 1 g of pullulan polysaccharide. PE containing Eu (2%, 4% and 6%) was then added to the mixture. In addition, 20% (gelatin *w*/*w*) glycerol was added as a plasticizer. The control was a pullulan–gelatin film containing essential oil emulsion. The film-forming solution was poured into the polymer mold with a diameter of 10 cm. After drying at 25 °C for 48 h, it was peeled off and stored at 53% relative humidity.

### 3.5. FT-IR

The FT-IR spectrometer (Thermo Nicolet Corporation, Madison, UK) was applied to assess the samples via the potassium bromide tablet pressing method. A potassium bromide slice was used as the blank control to collect the baseline. The composite film fragments were mixed with potassium bromide powder and pressed, and the data of the active film and Eu at 4000–500 cm^−1^ were collected by FT-IR [47].

### 3.6. FE-SEM

The film samples were pretreated and freeze-dried for 48 h. Firstly, conductive gold was precoated on the surface of the films using a sputtering coater (MODEL 682, Shanghai, China) in a vacuum, and the films were transferred to FE-SEM (SU8020, Hitachi, Japan) [7].

### 3.7. Thermogravimetric Analysis

The film (5 mg each sample) was put on the aluminum plate. The thermogravimetric analyzer was run over a temperature range of 30 °C to 600 °C with a heating rate of 10 °C/min, and the nitrogen was operated at the speed of 20 mL/min.

### 3.8. Characterization of Physical Properties of Films

#### 3.8.1. Thickness and Density of Films

Film thickness was measured with digital Vernier calipers using 10 random points, and the accuracy was 0.01 mm.

The following formula was used for density:

$$\rho =\frac{m}{A\times S}$$

where A—film area, cm^2^; S—film thickness, cm; m—film mass after conditioning, g; ρ—film density, g·cm^−3^.

#### 3.8.2. Measurement of Mechanical Properties

The tensile strength (TS) and elongation at break (E) were measured by a physical property meter (TA-XTplus, Stable, UK). Samples were dried at a relative humidity of 55% for 24 h. The initial spacing between the two probes was set as 40 mm and the stretching speed was set to 1 mm/s.

#### 3.8.3. Water Vapor Permeability

The water vapor permeability was determined following the previously described method [48]. Firstly, the film was cut into a disc shape (2 cm diameter) and sealed in a vial containing 3 g of anhydrous CaSO_4_ with a relative humidity of 0%. Then, the sealed bottle was transferred to a 25 °C thermostat filled with saturated K_2_SO_4_ solution, and the change in weight of the sealed bottle was measured every 24 h. The slope was calculated according to the linear regression equation (weight and time), so as to define the formula of water vapor transmittance:

$$\mathrm{WVP}=\frac{\mathrm{WVTR}\times d}{P\times (R_{1}-{\;R}_{2})}$$

where P—saturation vapor pressure of water at 25 °C, Pa; R_1_—RH in the desiccator, %; R_2_—RH in the sealed bottle, %; d—film thickness, m; WVP—water vapor permeability, g/m·H·Pa.

#### 3.8.4. Chromaticity of Films

The color index of the film sample was measure by a hand-held colorimeter (TS7700, 3nh, China), the values of L*, a* and b* were recorded, and the color difference, whiteness value, and yellow value of the film with corresponding values were characterized.

$$\Delta Ε=\sqrt{{(L*\;-\;L)}^{2}+(a*\;-\;a{)}^{2}+(b*\;-\;b{)}^{2}}$$

$$\mathrm{WI}=100\;-\sqrt{(100\;-\;L{)}^{2}+a^{2}+b^{2}}$$

$$\mathrm{YI}=\frac{142.86\times b}{L^{*}}$$

The films were cut to 20 mm in length and 5 mm in width, stacked inside the cuvette, and the light transmittance at 450 nm was measured.

### 3.9. Pretreatment of Chilled Beef

The beef samples were prepared on the basis of the optimized method previously presented by Zhang et al. [5]. Firstly, the cutting tools and other experimental equipment used in the experiment were disinfected and sterilized. The excess fat and tendons were removed from the fresh beef on a clean bench, and it was cut into blocks with uniform sizes and weights of about 15 g. Then the meat sample was divided into three groups in accordance with the package of composite films. They were stored in a refrigerator at 4 °C for 15 d. Their pH value, chromaticity, moisture content, TVB-N value, TBARS value and total colony value were determined at 0, 3, 7, 11 and 15 d. Three samples were taken from each treatment group.

### 3.10. Determination of pH Value

The pH value was measured following the procedures previously described [49]. Then, 5 g samples were randomly taken from the three groups of samples, crushed and washed with distilled water, then magnetically stirred for 20 min. Finally, 30 mL of supernatant was filtered, and its pH was determined with a precision pH meter.

### 3.11. Determination of Chromaticity

The sample was selected with a thickness of about 1.5 cm, and random points were viewed on the cross section with a colorimeter (TS7700, 3 nh, Shanghai, China) to determine the L* value, a* value and b* value of the beef. Each meat sample was repeated 3 times, and the mean value was taken as the chromaticity value.

### 3.12. Determination of Moisture Content

The moisture content of the beef sample was determined according to a previously described method [50], involving drying to constant weight in an oven at 75 ± 2 °C under direct heat. The moisture content was calculated by recording the weights of the film samples before and after evaporation.

### 3.13. Determination of Total Volatile Base Nitrogen (TVB-N) Value

The TVB-N values of beef samples were determined following the steps of a previous study [51]. The amount of total nitrogen in the aqueous leachate of beef samples that can be distilled with water vapor under alkaline conditions was determined using an automated Kjeldahl method.

### 3.14. Determination of Thiobarbituric Acid (TBARS) Value

Based on the method of Wei et al. [41] with slightly modifications, meat samples were mixed with trichloroacetic acid solution and homogenized for 60 s, and then centrifuged at 1750 g at 4 °C for 15 min. Then, the filtrate was mixed with 0.02 mol/L 2-thiobarbituric acid water, and transferred to a 90 °C water bath and heated for 30 min. For the blank group, 5 mL trichloroacetic acid solution was used and mixed with thiobarbituric acid water. After cooling to room temperature, the absorbance of the sample at 530 nm was measured and a standard curve was prepared.

### 3.15. Determination of Total Mesophilic Aerobic Bacteria (TMAB)

According to the method of Duran and Kahve [52], the TMAB test was designed for microbiological evaluation of the characteristics of packaged samples. TMAB counts were achieved in PCA after growth at 30 °C for 72 h.

### 3.16. Data Analysis

The data were analyzed using Duncan’ s multiple range tests through SPSS 24.0 at a significance level *p* < 0.05.

## 4. Conclusions

In this study, different concentrations of compound freshness-protecting films were prepared using Eu, whey protein isolate, inulin, gelatin, and pullulan as raw materials. The results show that the Eu-loaded emulsion showed good stability. After the addition of Eu to the films, some interactions between functional groups occurred, but did not significantly change the chemical structure of the pullulan–gelatin films. The incorporation of Eu-loaded PE significantly reduced the thickness of the films, and the presence of Eu led to a decrease in the water vapor transmission rate of the films, while the light scattering effect of Eu increased the light barrier properties. Eu is a better active ingredient than clove essential oil during the beef preservation process. During the 15 days of beef preservation, the Eu-containing composite film was able to slow down the increase in pH, water loss, and the rise of TVB-N. It effectively slowed down the color change of meat samples and the rate of beef fat oxidation, inhibited the growth and reproduction of microorganisms, delayed the active time of Eu, and extended the shelf life of beef. This study provides a reliable theoretical basis for the popularization and application of this film as a natural freshness preservation package for meat products at the industrial level. In the future, the general applicability of the film to other meat products will be further verified, and a more comprehensive analysis will be undertaken for the popularization and application of the film as a natural form of freshness preservation packaging for meat products.

## Figures and Tables

**Table 1:** Physical properties of different composite membranes.

Films	Thickness (μm)	Tensile Strength (MPa)	Elongation at Break (%)	Water Vapor Permeability (×10^−7^ g·m/m^2^·Pa·s)
Pure film	168.0 ± 3.29 ^a^	2.47 ± 0.21 ^a^	14.59 ± 1.03 ^a^	9.43 ± 0.32 ^a^
PE 0.2%	115.7 ± 5.16 ^b^	2.33 ± 0.15 ^ab^	13.81 ± 1.76 ^ab^	7.17 ± 0.46 ^b^
PE 0.4%	101.3 ± 6.52 ^c^	2.54 ± 0.17 ^c^	12.46 ± 1.29 ^b^	6.52 ± 0.33 ^c^
PE 0.6%	88.5 ± 2.33 ^d^	2.82 ± 0.25 ^d^	10.77 ± 0.81 ^d^	6.04 ± 0.18 ^c^

Different letters in the same column indicate significant differences among formulations (*p* < 0.05). Values are mean ± standard deviation (*n* = 3). The samples include pure film, 0.2% PE, 0.4% PE, and 0.6% PE, respectively.

**Table 2:** Chromaticity of beef preserved with a composite film for different times.

	Time	Control	Pure Film	0.2% PE	0.4% PE	0.6% PE
L*	0	45.92 ± 1.2 ^a,A^	45.92 ± 1.2 ^a,A^	45.92 ± 1.2 ^a,A^	45.92 ± 1.2 ^a,A^	45.92 ± 1.2 ^a,A^
	3	49.15 ± 1.5 ^ab,A^	48.32 ± 1.1 ^ab,A^	47.73 ± 0.7 ^ab,B^	47.37 ± 0.5 ^ab,B^	47.62 ± 0.8 ^ab,B^
	7	49.41 ± 1.7 ^ab,A^	48.68 ± 1.3 ^b,AB^	48.39 ± 0.5 ^b,AB^	48.06 ± 0.8 ^b,A^	47.97 ± 1.1 ^ab,A^
	11	49.03 ± 1.3 ^b,A^	48.82 ± 0.4 ^b,AB^	48.23 ± 1.0 ^b,B^	48.52 ± 1.1 ^b,AB^	48.34 ± 1.1 ^b,AB^
	15	48.74 ± 1.1 ^ab,A^	48.37 ± 0.8 ^ab,AB^	48.04 ± 1.2 ^a,AB^	48.33 ± 0.6 ^ab,AB^	47.73 ± 0.7 ^ab,B^
a*	0	2.87 ± 0.02 ^a,A^	2.87 ± 0.02 ^a,A^	2.87 ± 0.02 ^a,A^	2.87 ± 0.02 ^a,A^	2.87 ± 0.02 ^a,A^
	3	3.08 ± 0.05 ^b,A^	3.51 ± 0.03 ^b,B^	3.26 ± 0.07 ^b,C^	3.19 ± 0.02 ^b,D^	3.15 ± 0.06 ^b,D^
	7	3.37 ± 0.07 ^c,A^	4.08 ± 0.11 ^c,B^	3.70 ± 0.03 ^c,C^	3.81 ± 0.09 ^c,D^	3.64 ± 0.08 ^c,C^
	11	1.07 ± 0.02 ^d,A^	1.08 ± 0.03 ^d,A^	1.50 ± 0.04 ^d,B^	2.01 ± 0.03 ^d,C^	1.44 ± 0.03 ^d,B^
	15	0.78 ± 0.01 ^e,A^	0.89 ± 0.01 ^e,B^	1.00 ± 0.02 ^e,C^	1.30 ± 0.03 ^e,D^	1.05 ± 0.02 ^e,D^
b*	0	−4.08 ± 0.03 ^a,A^	−4.08 ± 0.09 ^a,A^	−4.08 ± 0.03 ^a,A^	−4.08 ± 0.03 ^a,A^	−4.08 ± 0.03 ^a,A^
	3	−3.57 ± 0.06 ^b,A^	−3.97 ± 0.06 ^a,B^	−3.86 ± 0.05 ^b,B^	−3.76 ± 0.06 ^b,C^	−3.77 ± 0.08 ^b,C^
	7	−3.60 ± 0.03 ^c,A^	−3.69 ± 0.07 ^b,AB^	−3.55 ± 0.04 ^c,AC^	−3.47 ± 0.03 ^c,C^	−3.60 ± 0.05 ^b,A^
	11	−2.72 ± 0.07 ^bd,A^	−2.83 ± 0.05 ^c,A^	−2.93 ± 0.03 ^d,B^	−2.68 ± 0.04 ^d,A^	−3.16 ± 0.07 ^c,D^
	15	−2.51 ± 0.02 ^b,A^	−2.62 ± 0.05 ^d,B^	−2.47 ± 0.04 ^e,A^	−2.92 ± 0.07 ^d,C^	−2.77 ± 0.05 ^d,D^

L* values are considered to be the brightness of the sample, and a* and b* values indicate color variation. The superscript lowercase letters represent significant difference (*p* < 0.05) (n = 3) within the same column. The superscript uppercase letters represent significant difference (*p* < 0.05) (n = 3) within the same row.

**Table 3:** Moisture content of composite film-preserved beef at different times.

	Time	Control	Pure Film	0.2% PE	0.4% PE	0.6% PE
Moisture content (%)	0	75.3 ± 1.2 ^a,A^	75.4 ± 1.7 ^a,A^	75.3 ± 1.1 ^a,A^	75.3 ± 1.4 ^a,A^	75.4 ± 1.6 ^a,A^
	3	74.5 ± 0.7 ^b,A^	73.2 ± 1.0 ^b,B^	73.5 ± 0.7 ^b,B^	73.8 ± 0.5 ^b,B^	73.3 ± 0.8 ^b,B^
	7	75.3± 1.5 ^a,A^	72.4 ± 1.8 ^b,B^	73.2 ± 0.9 ^b,C^	73.2 ± 0.8 ^bc,C^	72.9 ± 1.1 ^b,C^
	11	75.9 ± 0.9 ^b,A^	71.6 ± 1.4 ^b,B^	72.7 ± 1.5 ^b,C^	72.7 ± 1.1 ^bc,C^	72.3 ± 0.7 ^b,C^
	15	76.3 ± 1.1 ^c,A^	70.9 ± 2.1 ^b,B^	72.0 ± 1.6 ^b,C^	72.2 ± 0.7 ^c,C^	71.6 ± 0.9 ^b,C^

The superscript lowercase letters represent significant difference (*p* < 0.05) (n = 3) within same column. The superscript uppercase letters represent significant difference (*p* < 0.05) (n = 3) within the same row.

**Table 4:** Changes of total bacterial counts of beef preserved with composite films at different times.

	Time	Control	Pure Film	0.2% PE	0.4% PE	0.6% PE
TVC (Lg CFU/g meat)	0	4.25 ± 0.3 ^a,A^	4.25 ± 0.3 ^a,A^	4.25 ± 0.3 ^a,A^	4.25 ± 0.3 ^a,A^	4.25 ± 0.3 ^a,A^
	3	5.51 ± 0.2 ^b,A^	5.37 ± 0.1 ^b,A^	4.84 ± 0.1 ^b,B^	4.92 ± 0.1 ^b,B^	5.04 ± 0.2 ^b,C^
	7	8.12 ± 0.3 ^c,A^	7.66 ± 0.3 ^c,B^	7.12 ± 0.2 ^c,C^	7.01 ± 0.2 ^c,C^	6.98 ± 0.4 ^c,C^
	11	9.43 ± 0.5 ^c,A^	9.35 ± 0.1 ^d,A^	8.97 ± 0.4 ^d,B^	8.61 ± 0.1 ^c,B^	8.34 ± 0.2 ^c,C^

The superscript lowercase letters represent significant difference (*p* < 0.05) (n = 3) within same column. The superscript uppercase letters represent significant difference (*p* < 0.05) (n = 3) within same row.

## Data Availability

The data used to support the findings of this study can be made available by the corresponding author upon request.

## Sample Availability

Not available.
